# Can First-Dose Therapeutic Drug Monitoring Predict the Steady State Area Under the Blood Concentration-Time Curve of Busulfan in Pediatric Patients Undergoing Hematopoietic Stem Cell Transplantation?

**DOI:** 10.3389/fped.2022.834773

**Published:** 2022-04-07

**Authors:** Abdullah Alsultan, Ahmed A. Albassam, Abdullah Alturki, Abdulrahman Alsultan, Mohammed Essa, Bader Almuzzaini, Salman Alfadhel

**Affiliations:** ^1^Department of Clinical Pharmacy, College of Pharmacy, King Saud University, Riyadh, Saudi Arabia; ^2^Clinical Pharmacokinetics and Pharmacodynamics Unit, King Saud University Medical City, Riyadh, Saudi Arabia; ^3^Department of Clinical Pharmacy, Prince Sattam Bin Abdulaziz University, Al-Kharj, Saudi Arabia; ^4^Pharmaceutical Analysis Lab-King Abdullah International Medical Research Center, Riyadh, Saudi Arabia; ^5^Department of Pediatrics, College of Medicine, King Saud University, Riyadh, Saudi Arabia; ^6^Oncology Center, King Saud University Medical City, Riyadh, Saudi Arabia; ^7^Department of Pediatric Hematology/Oncology, King Abdullah Specialist Children Hospital, Riyadh, Saudi Arabia; ^8^College of Medicine, King Saud bin Abdulaziz University for Health Sciences, Riyadh, Saudi Arabia; ^9^King Abdullah International Medical Research Center, National Guard Health Affairs, Riyadh, Saudi Arabia; ^10^Medical Genomics Research Department, King Abdullah International Medical Research Center, Ministry of National Guard Health Affairs, Riyadh, Saudi Arabia

**Keywords:** busulfan, pharmacokinetics, TDM (therapeutic drug monitoring), Bayesian pharmacokinetics, area under the blood concentration-time curve (AUC)

## Abstract

Busulfan has high intra-individual variability and possible time-dependent changes in clearance, which complicates therapeutic drug monitoring (TDM), as first dose sampling may not predict the steady state concentrations. In this study, we aimed to use Bayesian pharmacokinetic parameters estimated from the first dose to predict the steady state AUC for busulfan. This observational study was conducted among pediatric patients at King Abdullah Specialist Children’s Hospital. From each patient, we collected six blood samples (2, 2.25, 2.5, 3, 4, and 6 h after the start of IV infusion of the first dose). A subset of patients were also sampled at the steady state. First, we modeled the data using only the first dose. The model was used to estimate the empirical Bayesian estimates of clearance for each individual patient, then we used the empirical Bayesian estimates of clearance to predict the AUC_0–tau_ at steady state (i.e., predicted AUC_0–tau)_. Steady state AUC_0–tau_ was also calculated for patients sampled at steady state using the trapezoidal method using raw time concentration data; this was considered the reference AUC_0–tau._. Then, we compared the AUC_0–tau_ predicted using the Bayesian approach with the reference AUC_0–tau_ values. We calculated bias and precision to assess predictability. In total we had 33 patients sampled after first dose and at steady state. Using the Bayesian approach to predict the AUC_0–tau_, bias was −2.8% and precision was 33%. This indicates that first dose concentrations cannot accurately predict steady state busulfan concentrations; therefore, follow-up TDM may be required for optimal dosing.

## Introduction

Busulfan is an alkylating drug used in clinical practice as a component of conditioning regimens for patients undergoing hematopoietic stem cell transplantation (HSCT). The initial intravenous dose of busulfan for pediatric patients undergoing HSCT is usually given as 2-h infusions every 6 h over 4 days. The US FDA labeling recommends that the initial dose is based on body weight. The recommended dose is 1.1 mg/kg for patients ≤ 12 kg and 0.8 mg/kg for patients ≥ 12 kg (1). However, even when using weight-based dosing, a significant proportion of patients fail to achieve the therapeutic target, which increases the risk of treatment failure and toxicities (2–4).

Busulfan has a narrow therapeutic window, and wide inter and intra-patient pharmacokinetic variation is observed (3, 5, 6). Serious side effects such as veno-occlusive disease (VOD), neurotoxicity, acute graft vs. host disease, and even death have been linked to higher AUCs for this drug. On the other hand, lower AUCs are associated with disease relapse, unsuccessful engraftment, and poorer survival rates (7, 8). Therefore, several centers perform therapeutic drug monitoring (TDM) to optimize busulfan dosing (9, 10). The suggested target ranges to maximize efficacy and minimize toxicity are cumulative AUC_0–96_ values of 59.2–88.8 mg.h/mL (11) or, more recently, 78–101 mg.h/mL (8).

TDM is usually performed after a test dose or the first dose (12, 13). However, the high intra-individual variability and possible time-dependent changes in clearance (Cl) during elimination may limit the validity of TDM for busulfan after the first dose (6). Several studies demonstrated busulfan has time-dependent activity—with some studies reporting increased Cl, while others reported decreased Cl (1–4). Additionally, other studies described very high intra-individual variability (5, 6). Regardless if its random intra-individual variability or systematic effects due to time-dependent changes in Cl, the important clinical question is whether we can use TDM data from the first dose to guide dose adjustment. In this study, we aimed to evaluate the predictive value of Bayesian pharmacokinetic parameters estimated from the first dose to predict the steady state AUC for busulfan in pediatric patients.

## Methods

### Study Design and Sample Collection

This observational study was conducted at King Abdullah Specialist Children’s Hospital in Riyadh, Saudi Arabia. The inclusion criteria were: pediatric patients (0–14 years) undergoing allogeneic hematopoietic stem cell transplantation receiving busulfan as part of their conditioning regimen. Busulfan was administered as a 2-h intravenous infusion every 6 h for 4 days. The protocols for sample collection and the analytical assay were as described in our prior publication (14). In summary, we collected six blood samples at 2, 2.25, 2.5, 3, 4, and 6 h after start of IV infusion of the first dose). Blood samples were centrifuged and plasma was stored at −70°C until the day of analysis.

A subset of patients were also sampled at steady state, which is usually after dose number 5. Sampling at steady state is usually performed for patient with low or high busulfan concentrations. Data included in this study includes the 59 patients from our prior publication (14).

### Analytical Assay

Busulfan was analyzed using a validated method, as previously described (14). In summary, chromatographic separation was conducted using a Water Acquity UPLC^®^ followed by detection on a Xevo TQ-MS A tandem quadrupole LC-MS/MS (Milford, MA, United States). The mobile phase was composed of 55% de-ionized water, 20% methanol, and 25% acetonitrile containing 250 μL acetic acid, 250 μL ammonia solution (25%), and 100 μL formic acid in order to enhance ionization and stabilization. The mobile phase was delivered at a flow rate of 0.30 mL/min. The temperature column was set to 30°C and the injection volume was 10 μL. The analytes were detected in positive ionization mode, ESI+, using multiple reaction monitoring (MRM), using a *m/z* transition at 264.1/151.0 for busulfan and 271.2/91.0 for the internal standard. Data were processed using Mass Lynx Software version 4.1. for system-control, data acquisition, and data processing. Linearity of the assay was achieved for concentrations ranging between 25 and 1,600 ng/mL; the correlation coefficient was 0.99. The intra-day and inter-day precision and accuracy of the assay were within the values recommended in the FDA bioanalytical method validation guidance.

### Ethical Approval

The study was approved by the local Institutional Research Ethics Board at King Abdullah International Medical Center.

### Genotyping

A subset of our patients were genotyped for *GSTA1*, *GSTP1*, and *GSTM1* to assess the impact of these variations on busulfan pharmacokinetics and/or time dependent changes in busulfan Cl. DNA was extracted from the blood samples using PureLink™ Genomic DNA Mini Kit. We aimed to identify single nucleotide polymorphisms (SNPs) in different genes including *GTSA1*, *GSTP1*, and *GSTM1.* For the region holding the *GSTA1* Gene we investigated SNP ID rs1131965, for *GSTP1* we investigated SNP ID rs8191448 and for *GSTM1* we investigated SNP ID rs2071487. Data analysis was performed using TaqMan genotype software.

### Population PK Model

First, we modeled the data using the time concentration profiles for the first dose only. We used a one-compartment model with linear elimination parametrized in terms of Cl and V. Only bodyweight was added as a covariate for both Cl and V. Weight was scaled to 20 kg and we used exponents of 0.75 for Cl and 1 for V, as in our prior model. The model was evaluated using standard goodness of fit plots and we assessed the shrinkage and relative standard error of the pharmacokinetic parameter estimates. Generally, shrinkage values should be <20% for empirical Bayesian estimates. The model developed was used to estimate the empirical Bayesian estimates of Cl. Population pharmacokinetic modeling was performed using Monolix 2020 (Lixoft, France).

### Calculation of Bayesian AUC_0–tau_

For each individual patient, we used the empirical Bayesian estimates of Cl to predict the AUC_0–tau_ at steady state, in which AUC_0–tau_ was considered as the predicted AUC_0–tau_


A⁢U⁢C=D⁢o⁢s⁢e/C⁢l

### Calculation of AUC_0–tau_ Using Non-compartmental Analysis

AUC_0–tau_ at steady state was calculated for patients sampled on two occasions (first dose and steady state) via the trapezoidal method using the raw time concentration data on occasion 2. This value was considered as the reference or true AUC_0–tau_. Non-compartmental analysis was performed using PKanalix. At our center, occasion 2 indicates sampling at dose number 5 or later.

### Statistical Analysis

For each patient, we compared the AUC_0–tau_ predicted using the Bayesian approach with the reference AUC_0–tau_. To assess predictability, we calculated bias (mean prediction error) and precision (root mean squared error), as follows (15):


$$B\,i\,a\,s\%=\frac{\Sigma \,\left(A\,U\,C\,p\,r\,e\,d\,i\,c\,t\,e\,d-A\,U\,C\,r\,e\,f\,e\,r\,e\,n\,c\,e\right)}{N}*\left(\frac{100}{A\,U\,C\,m\,e\,a\,n}\right)$$

$$P\,r\,e\,c\,i\,s\,i\,o\,n\%=\sqrt{{\frac{\Sigma \,\left(A\,U\,C\,p\,r\,e\,d\,i\,c\,t\,e\,d-A\,U\,C\,\text{reference}\right)}{N}}^{2}}*\left(\frac{100}{A\,U\,C\,m\,e\,a\,n}\right)$$

For good prediction, bias should be <5% and precision, <20%. Bland Altman plots were also used to assess the agreement between the predicted and observed AUC_0–tau_.

Continuous covariates are presented as mean ± standard deviation, categorical variables are presented as percentages. All statistical analysis was performed using R statistical software and Microsoft Excel.

## Results

### Patients

Between January 2016 and June 2020, our center performed TDM for 127 pediatric patients who received busulfan via IV infusion as part of a conditioning regimen for HSCT. The total number of samples was 762. Average ± SD age was 6.5 ± 3.6 years, average ± SD weight was 21.5 ± 10.5 kg, and average ± SD dose received was 1 ± 0.13 mg/kg. We also obtained samples at steady state for 33 patients. Among these 33 patients, no dose adjustments were made for four patients, the dose was decreased for six patients, and the dose was increased for 23 patients. The baseline demographics of the patients sampled at steady state are shown in Table 1.

**TABLE 1:** Baseline demographics.

	Full data set *N* = 127	Patients sampled on 2 occasions (dose 1 and at steady state) *N* = 33
Age	6.4 ± 3.6	5.88 + 4.25
Weight	21.4 ± 10.7	21.64 + 14.19
Gender	Male = 60 Female = 67	Male = 19 Female = 14
Dose (mg/kg)	1 ± 0.14	1.00 + 0.166

*Data presented as mean + sd.*

### Genotyping

A total of 69 pediatric patients were genotyped for *GSTA1*, *GSTP1*, and *GSTM1* to assess the influence of genetic polymorphisms on busulfan pharmacokinetics. None of the patients had a genotype known to be associated with a reduction in busulfan clearance; therefore, comparison of pharmacokinetics between genotype groups was not possible. In our analysis, 62 patients (89.9%) were carriers of GSTA1*A/*A, seven patients (10.1%) were carriers of GSTA1*A/*B, and no patients were carrying GSTA1*B/*B. All 69 patients were carrying GSTP1*A/*A. A total of 42 of patients were GSTM1 positive and 27 patients were neither GSTM1 positive or null; these patients could be carrying different alleles that are not detected by the genotyping assay. Previous studies reported that busulfan clearance is reduced among patients carrying GSTA1*B/*B, GSTM1-null, and GSTP1*A/*G (16).

### Population PK

**FIGURE 1:**
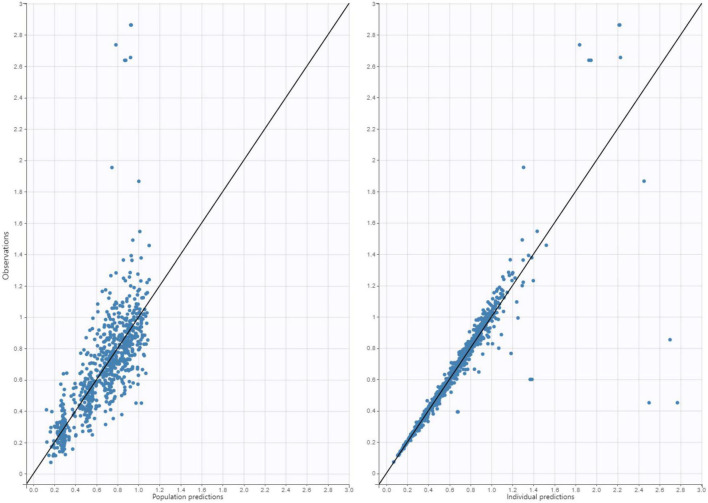
Goodness-of-fit plot for final population pharmacokinetic model. Right: Individual predictions of busulfan vs. observed concentrations. Left: Population predictions of busulfan vs. observed concentrations.

As a one-compartment model adequately described the data, a proportional error model was used to describe the residual variability. The PK parameter estimates (scaled to 20 kg) were Cl = 5.02 L/h and V = 16.1 L (0.8 L/kg). The estimated exponents were 0.83 (RSE% = 6.4%) for V and 0.76 (RSE % = 6.1%) for Cl, relatively close to 1 and 0.75 (17). Therefore, to simplify the model, the exponents were fixed at 1 for V and 0.75 for Cl. RSE% was low for PK estimates (Table 2). The concentrations predicted for each individual were in good agreement with the observed concentrations (Figure 1). Shrinkage was low for both Cl and V (<3%), indicating empirical Bayesian estimates of Cl can be used to predict the AUC for individual patients.

**TABLE 2:** PK parameter estimates.

	Base model	Final model
PK parameter	Estimate (RSE%)	Estimate (RSE%)
V (L)	15.17 (4.63%)	16.1 (2.73%)
BSV for V	51% (6.46%)	29.6% (6.8%)
Shrinkage for V	0.7%	0.9%
Cl (L/h)	4.78 (4.34%)	5.02 (2.52%)
BSV for Cl	48% (6.44%)	27.8% (6.6%)
Shrinkage for Cl	1.74%	2.9%
Residual variability (b)	0.11 (3.16%)	0.1 (3.17%)

*RSE, relative standard error; BSV between subject variability expressed as the coefficient of variation %.*

*Both V and Cl in the final model are scaled to 20 kg.*

*V = 16.1 × (weight/20).*

*Cl = 5.02 × (weight/20)^0.75^.*

### AUC_0–tau_ Predictions and Bayesian Forecasting

**FIGURE 2:**
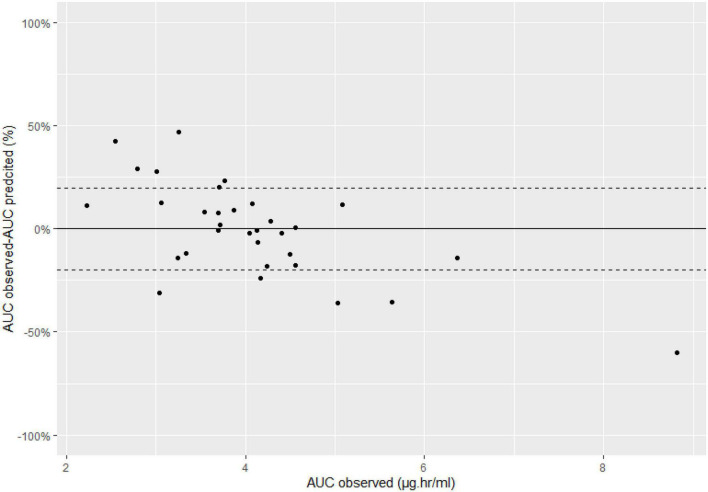
Bland-Altman plots, dashed lines represent 20%.

In total, we sampled 33 patients after both the first dose and at steady state (Table 1). Using the Bayesian approach to predict the AUC_0–tau_, the bias was −2.8% and precision was 33%. AUC was over predicted in 17 patients (51%) and under predicted in 15 patients (49%). Of the 33 patients, 12 patients (36%) had a predicted AUC_0–tau_ outside the range of ±20%. Moreover, predictability was very poor (>50% error) for four patients; the AUC_0–tau_ was under predicted in three of these patients and over predicted in one patient (Table 3). Figure 2 shows the Bland Altman plot.

**TABLE 3:** Bias and precision for AUC predictions.

	Bayesian approach
Bias	−2.8%
Precision	33%
% Error > 20%	12
% Error > 50%	4

## Discussion

Based on our analysis, using the time concentration profile after the first dose does not necessarily accurately predict the steady state concentration of busulfan in pediatric patients. This indicates significant intra-individual variability exists and there is a need for follow-up TDM in pediatric patients treated with busulfan; we suggest that TDM should be performed repeatedly over 4 days or, at a minimum, twice after the first dose and at steady state. More than one third of our patients had an AUC either 20% higher or lower than predicted. In our analysis, we used non-compartmental analysis to predict the steady state AUC. This is the most common method used to do TDM for busulfan. Another approach to estimate the steady state AUC is to use the empirical Bayesian estimates from dose 1. We did test that approach and got similar results to the non-compartmental analysis method (bias was −1.1% and precision was 35%).

Busulfan is a chemotherapeutic drug with a narrow therapeutic window, thus such differences could increase the risk of toxicity or treatment failure. A limitation of follow up TDM, is the additional burdensome on the patient, hospital staff and lab personnel. TDM of busulfan requires taking 5–7 samples per dosing occasion to estimate the AUC. An alternative approach is to utilize the concept of limited sampling strategy. Several prior publications have developed limited sampling strategies to estimate the AUC using 1–3 samples. These studies used a mix of both Bayesian (parametric and non-parametric) and linear regression approaches and can be easily implemented in clinical practice (10, 18–20).

Overall, our model and parameter estimates are consistent with previous studies. Most prior busulfan pharmacokinetics models used a one compartment model with linear elimination. For the effect of covariates, almost all studies included bodyweight for both Cl and V, while some also included age. The estimated V and Cl scaled to a 20 kg patient in these studies ranges from 12 to 20 L and 4–9 L/h, similar our results (6, 19, 21, 22). A major concern with busulfan and where several prior studies report mixed results is the high intraindividual variability with some showing an increase and others, a decrease, in Cl over time (6, 10). This intraindividual variability could be random, or systematic due to time-dependent changes in Cl or a combination of both. Hassan et al. assessed five adult patients with acute myeloblastic leukemia who received oral busulfan; for all patients, the observed steady state AUC_0–tau_ was lower than predicted. The half-life was 3.4 h after the first dose and 2.3 h after the last dose (23). In another study by Hassan et al. that included both adult and pediatric patients, about 35% of participants showed 30–60% lower steady state concentrations than predicted, while none showed a higher steady state concentration (24). Lindley et al. included both adult and pediatric patients who received oral busulfan, and found total body clearance increased from 9,247 mL/h after the test dose to 11,218 mL/h after dose number 13. In our prior study, 9 of 15 patients had lower than predicted AUC_0–tau_ at steady state (14). Some other studies have reported the opposite results, in that Cl decreases over time (25–27). Long-Boyle et al. reported Cl decreases by approximately 20% over time (25). Gaziev et al. found Cl was 20% higher after the first dose than later doses (26). Marsit et al. evaluated the intra-individual (also sometimes denoted as inter-occasion) variability in Cl in a large cohort of pediatric patients (*n* = 136) sampled on multiple occasions, and observed both large increases and decreases in busulfan Cl. Most patients in their study exhibited decreased busulfan Cl, though the authors could not identify any factors that accurately predict whether Cl will increase or decrease (6).

Based on these prior studies, it is clear busulfan has high intra-individual variability. However, it is not known if these variations are systematic or both systematic and random. Possible factors could include pathophysiologic changes during treatment (such as hepatic dysfunction), genetic factors, glutathione depletion, drug interactions, and inflammation (which is associated with downregulation of liver enzymes). In terms of genetic effects, most of our patients carried the wild-type alleles; therefore, we could not evaluate whether genotype influences the predictive value of first dose sampling in our cohort. For glutathione, a previous semi-mechanistic model suggested that the time dependent decrease in busulfan Cl is due to glutathione depletion. Patients with relatively high initial Cl showed a more pronounced reduction in Cl at steady state (28). Future studies that evaluate the pharmacokinetics of busulfan and measure glutathione concentration can help answer the some of the questions related to busulfan time dependent clearance.

The limitations of our study include the small number of patients sampled at steady state, which reduced the power to identify factors associated with increased/decreased Cl. Nevertheless, Marsit et al. could not identify any significant predictors of changes in Cl in a larger cohort of patients (6).

In conclusion, combined with other studies, this study demonstrates that high intra-individual variability is observed for busulfan. Therefore, follow up TDM is recommended for optimal dosing in pediatric patients. Additional studies are needed to further delineate the causes of this variability, which are likely to be multifactorial.

## Data Availability Statement

The original contributions presented in the study are included in the article/supplementary material, further inquiries can be directed to the corresponding author.

## Ethics Statement

The studies involving human participants were reviewed and approved by the Local Institutional Research Ethics Board at King Abdullah International Medical Center, Riyadh, Saudi Arabia (IRB # RC17/131/R). Written informed consent to participate in this study was provided by the participants’ legal guardian/next of kin.

## Author Contributions

AAls and AAA: manuscript writing and pharmacokinetic analysis. AAlt: manuscript reviewing and analytical assay. ARAls and ME: manuscript writing, study design, and patient recruitment. BA: manuscript writing and genotyping. SA: manuscript reviewing, study design, and analytical assay.

## Conflict of Interest

The authors declare that the research was conducted in the absence of any commercial or financial relationships that could be construed as a potential conflict of interest.

## Publisher’s Note

All claims expressed in this article are solely those of the authors and do not necessarily represent those of their affiliated organizations, or those of the publisher, the editors and the reviewers. Any product that may be evaluated in this article, or claim that may be made by its manufacturer, is not guaranteed or endorsed by the publisher.
